# Social Context and Tool Use Can Modulate Interpersonal Comfort Space

**DOI:** 10.3390/jcm12041647

**Published:** 2023-02-18

**Authors:** Antonella Ferrara, Mariachiara Rapuano, Gennaro Ruggiero

**Affiliations:** Laboratory of Cognitive Science and Immersive Virtual Reality, CS-IVR, Department of Psychology, University of Campania “Luigi Vanvitelli”, 81100 Caserta, Italy

**Keywords:** reaching-comfort distance, action-social space, tool-use, motor plasticity, social context

## Abstract

Recent research has investigated whether the representation of space around the body, in terms of reach–action (imagining of reaching another person) and comfort–social (tolerance of the other’s proximity) spaces, may reflect a shared sensorimotor basis. Some studies exploiting motor plasticity induced by tool use have not observed sensorimotor identity (i.e., the same mechanisms that underlie, based on sensory information, the representation of proximal space in terms of action possibilities, goal-directed motor actions, and anticipation of the sensorimotor consequences), whereas evidence to the contrary has also emerged. Since the data are not fully convergent, here we wondered whether or not the combination of motor plasticity induced by tool use and the processing of the role of social context might reflect a similar modulation in both spaces. To this end, we conducted a randomized control trial with three groups of participants (N = 62) in which reaching and comfort distances were measured in Pre- and Post-tool-use sessions. The tool-use sessions were conducted under different conditions: (i) in the presence of a social stimulus (determining the social context) (Tool plus Mannequin group); (ii) without any stimulus (Only Tool group); (iii) in the presence of a box (Tool plus Object group) as a control condition. Results showed an extension of comfort distance in the Post-tool session of the Tool plus Mannequin group compared with the other conditions. Conversely, the reaching distance was larger after tool use than at the Pre-tool-use session, independently of the experimental conditions. Our findings suggest that motor plasticity impacts reaching and comfort spaces to different degrees; while reaching space is markedly sensitive to motor plasticity, comfort space needs qualification of social context information.

## 1. Introduction

The space that surrounds the body is the area where all physical interactions between the individuals and the external stimuli can occur. In proxemics, this space is called interpersonal space [1,2,3] and represents the space that people maintain to interact with conspecifics (measured as comfort distance) (see [4,5]). Indeed, a typical task to assess the size of interpersonal space is based on comfort distance (“stop-distance paradigm”), in which participants stop the interactants at the point where they still feel comfortable with their proximity [1,2,3,6,7,8,9]. 

In neuroscience, the term peripersonal space defines the multisensory interface of the representation of the space closely surrounding the body [10,11,12,13,14] (for reviews, see [15,16]). The term is also commonly used to define the portion of space within the reach of our limbs [13,17,18]. It is important to note that here we are referring to the portion of reachable space (e.g., [19,20,21]). How we interact with the environment surrounding us is based on the information processed by our brain on the body and the space in which it is embedded. Indeed, the brain constantly processes a functional representation of the environment, i.e., the result of the integration of information from the current sensory-motor state and previous interactions with the physical and social context [1,22,23]. Evidence showed that while the reaching space is processed in the fronto-parietal network (including the cerebellum), the extrapersonal space (out of reach) information is mainly processed through the ventral visual stream [24]. A useful task to assess the size of the reaching space is to ask people to judge whether stimuli presented at various distances from the body are reachable or not (e.g., [25]). In general, people are able to determine the extension of their reachable space in relation to the length of their arm (e.g., [26,27]).

Research has questioned whether there is a relationship between the space to act on objects and the space to interact with conspecifics (see [4,5]). This is largely because points of contact between the two spaces have been observed (e.g., common defensive mechanisms according to the safety/threat value of contextual factors [28,29,30,31,32]). More specifically, evidence showed that the space around the body is influenced by interaction with social stimuli [28,29,30]. Several behavioral studies also reported similarities between reaching and comfort spaces that may reflect, albeit to different degrees, common sensorimotor mechanisms when combined with the processing of the social-emotional valence of stimuli (e.g., [5,31]). Studies in virtual (e.g., [5,31,32]) and real-world [33] contexts compared the sizes of reaching (i.e., distance at which people perceive a stimulus as reachable) and comfort (i.e., distance people prefer from other persons) distances in different approach conditions (see [1,2,6,7,34,35,36,37]). Overall, the size of both spaces was similarly modulated by the socio-emotional context: reduced in the interaction with humans compared to with objects [5,31,33,38]. The functional similarity between the reaching and comfort spaces was also observed under other experimental conditions: both were larger when dealing with confederates described as immoral [38] (see also [29]), showing angry facial expressions [31], or under unpleasant perceived temperatures [32]. 

Recently, in the wake of the above findings, some research has wondered whether the two spaces share a sensorimotor identity (i.e., the same mechanisms that underlie, based on sensory information, the representation of proximal space in terms of action possibilities, execution of goal-directed motor actions, anticipation of sensorimotor consequences) [39,40,41]. To this end, these studies have exploited the sensorimotor plasticity of the reaching space induced by the use of a tool (e.g., [11,42,43,44,45,46,47]). Specifically, Patané and colleagues [39], using the same task devised by Iachini and colleagues [5], compared reaching and comfort distances before and after a tool-use training session. The rationale behind was that if the two spaces share the same mechanisms, any sensorimotor plastic change induced in the reaching representation, as indexed by the reaching-distance task, should modulate the comfort one, as indexed by the comfort-distance task. The results showed a dissociation: tool use remapped the reaching distance, but not the comfort distance. This conclusion was corroborated by a subsequent study with a cooperative social context [40]. Participants completed reaching/comfort tasks toward a confederate before and after using a (long vs short) tool in cooperative and non-cooperative conditions. Overall, the results showed that comfort distance was affected by the social cooperative context and not by the length of the tool, whereas reaching distance was affected by the use of the long tool independently of the kind of the social interaction. They concluded that the two spaces rely on dissociable plastic mechanisms. In contrast, Quesque and colleagues [41], by simulating an interaction in a social context with an approaching human-like point-light displays, assessed the comfort space before and after a tool-use session. They found an increase in the minimum comfort distance after tool use, suggesting that the modulation of the comfort space was related to the reaching space. 

So far, evidence does not seem to converge and there are still unexplored aspects. While on the one hand the sensorimotor component appears distinguishable, on the other hand the weight of information processing coming from the social context seems less clear. With regard to Quesque et al. [41], for example, it could be argued that the two spaces have not been directly compared. Instead, in the studies of Patané and colleagues [39,40], some critical aspects may lie in the effect of familiarity of the confederate used and cooperative conditions. As for the latter, in proxemics it is known that repeated encounters with the same person can cause a reduction in comfort distance due to an increase in the sense of familiarity [48,49,50,51]. As for the cooperative context, participants might have taken into account the portion of space of the other, trying not to invade it (e.g., [52,53]). This would be also in line with previous observations in which people tend to assign specific regions of the workspace to one another in cooperative motor tasks by adapting their behaviour to fit the social context [54,55].

Building on this, the aim of this study was to clarify to what extent the processing of information from the social context combined with motor plasticity induced by tool use may or may not produce similar sensitivity to reaching and comfort space. To this end, between two blocks (Pre-Post session) of reaching and comfort tasks, three groups of participants performed tool-use training sessions under the following conditions: (i) tool-use training in a social context, i.e., in the presence of the mannequin; (ii) tool-use training without any kind of stimulus; (iii) tool-use training in the presence of a large box (as control condition). Finally, to control the effect of familiarity and thus maintain a similar level of tolerance of proximity to the other, two confederates were involved in the Pre-tool-use session and two different confederates were involved in the Post-tool-use session.

We hypothesized a larger comfort distance after tool use only in the social context condition and we expected a larger reaching distance after tool use in all three groups. To test our hypotheses, we conducted two kinds of analysis: two ANOVAs considering the mean Pre-tool-use and Post-tool-use distances per each task (reaching and comfort) and an ANOVA considering the mean differential distances (i.e., Post-tool-use distance minus the Pre-tool-use distance) obtained for each task. The rational was as follows: positive values are indicative of elongation of the space after training with the tool, negative values are indicative of contraction of the space after training with the tool. 

Finally, since it is widely accepted in the proxemics literature that male dyads maintain larger distances than female dyads (e.g., [33,56]), we wondered whether sex differences could also appear in the participant–confederate interaction. Overall, a smaller distance with female than male confederates should emerge.

## 2. Materials and Methods

### 2.1. Participants 

Sixty-two subjects (thirty females) aged 19–30 years (M = 22.7; SD = 3.0) took part in this experiment in exchange for course credit. Participants were randomly assigned to the three experimental conditions: Only Tool (OnlyT), Tool plus Mannequin (TM) and Tool plus Object (TObj). More specifically, 21 subjects (10 females) aged 20–30 years (M = 23.7, SD = 3.5) were recruited in the OnlyT group; 21 subjects (9 females) aged 20–30 years (M = 23.9, SD = 5.07) were included in the TM group, and 20 subjects (11 females) aged 19–25 years (M = 20.5, SD = 1.5) were assigned to the TObj group. Participants were naïve to the experimental hypotheses and the task used and had no self-reported history of neurological or psychiatric diseases. All subjects had normal or corrected to normal vision. Notably, participants and confederates involved in the study were unknown to each other and they could not interact during the experimental sessions. All the participants provided written informed consent to participate in the experiment, which was approved by the Ethical Committee of the University of Campania “L. Vanvitelli”, in agreement with the 2013 Helsinki Declaration [57].

### 2.2. Setting

The experiment was carried out in a sound-proofed room of the Laboratory of Cognitive Science and Immersive Virtual Reality (CS-IVR), Dept. Psychology (University of Campania Luigi Vanvitelli, Caserta, Italy). The experimental setting consisted of a room (5 m × 4 m × 3 m) with a three-meter path, marked on the floor with adhesive tape and a table (100 cm × 60 cm) with two chairs at each end. The table was used in the tool-use training session.

### 2.3. Materials

A 75 cm grabber was used for the tool-use training session. Participants were asked to reach and retrieve three different geometrical objects (large, medium, and small) placed beyond the reachable distance of the subjects (≈80 cm from the participant’s sternum). The distance between the participant and the confederate, during the reaching/comfort tasks, was recorded with a digital laser meter (Agatec, model DM100, error ± 0.003 m). 

In the TM group, during the tool-use training session, an anthropomorphic mannequin with the appearance of a female young adult was used. The mannequin had a neutral expression and wore black clothes (as the confederates we involved). Based on previous studies on the effect of mixed and same-sex dyads on proxemics (see [33]), we preferred to use a mannequin with the appearance of a female young adult to minimise the potential impact on the outcome due to the sex differences of participants. In the TObj group, during the tool-use training session, a large grey box was used.

### 2.4. Procedure

All participants were first instructed on the tasks and the tool-use training session and then led to a predetermined position. There, participants received a training session about the distance judgment tasks with the help of a confederate not involved in the testing phase. Each experimental session comprised a first set of distance judgments, followed by the tool-use training and ending with a second set of distance judgments. Participants provided the reaching-distance judgments (Instruction: “stop the confederate at the distance you think you can reach him/her”) and the comfort-distance judgments (Instruction: “stop the confederate at the distance you feel uncomfortable with him/her proximity”) by saying aloud “Stop”. The testing phase began with the participant standing on the predetermined position at the head of the three-meter path marked on the floor and while a confederate stood at the other head. To avoid any effect due to the familiarization during the approach, four confederates (two males and two females) were recruited in this study. Previous pilot studies revealed that repeated human–human interactions can induce a contraction of social boundaries. On this basis, two confederates, (one male and one female) were presented in the Pre-tool-use session and two more confederates (one male and one female) were introduced in the Post-tool-use session. Moreover, to avoid any effect due to aesthetic or idiosyncratic characteristics, all confederates wore black clothes, were instructed to keep a neutral facial expression, and fix the gaze at the height of the subject’s forehead by avoiding direct eye contact [4]. Confederates adopted the same walking speed (about 0.5 m/s) throughout the experimental sessions. During the two reaching/comfort tasks, participants stood still and saw the confederates walking towards them. Immediately after stopping the confederate, participants had to close their eyes and then the distance from the confederate was measured. More specifically, the distance between the confederate’s chest and the participant’s chest was measured by a digital laser meter (Agatec, model DM100, error ± 0.003 m). Participants had to determine four reaching distances and four comfort distances before, and four reaching distances and four comfort distances after tool use (two with each male and female confederate), for a total of 16 trials. The order of tasks was counterbalanced across participants. The order of presentation of the confederates was also counterbalanced across all conditions and participants.

As regard the tool-use training session, the subjects seated at the table, were asked to reach and retrieve one at a time the objects (tot = 96) located out of their reachable space using a 75 cm long grabber. The stimuli were randomly placed at different azimuthal and radial locations, in order to cover the entire participant’s action space. Participants were instructed to carry out a continuous and fluid movement to reach and retrieve the objects and to be as accurate as possible. The tool-use session was the same for all groups. 

**Figure 1:**
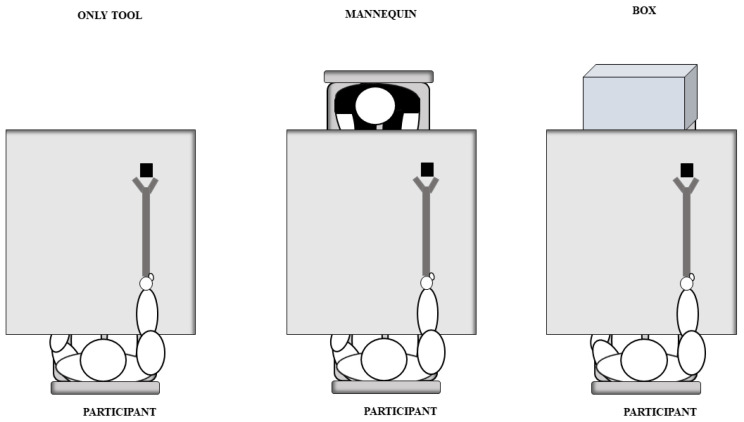
The three different tool-use training conditions. From left to right in the figure is shown the Only Tool condition (OnlyT group) in which participants performed the training in absence of stimuli, the Tool plus Mannequin condition (TM group) in which participants performed the training in a social context that is in presence of mannequin, and the Tool plus Object condition (TObj group) in which participants performed the training in front of a large box.

In the TM group, participants performed the tool-use training in a social context, that is, in the presence of a mannequin seated in front of them at the end of the table (see Figure 1). According to the literature on human–human and human-like interactions (e.g., human–robot interactions), anthropomorphic characteristics are sufficient for a stimulus to be socially connoted and treated more like a real person than an object (e.g., [30,58,59]). In the TObj group, participants performed the tool-use training in the presence of a large box placed at the end of the table (see Figure 1). In the OnlyT group, participants performed the training without any kind of stimulus in front of them (see Figure 1). At the end of the testing phase, there was a post-experimental debriefing in which participants were asked to rate their sense of familiarity with the confederates in both Pre-/Post-tool-use interactions. They were also asked to report on their experience with the confederates and whether they had noticed anything special. As for TM group, participants were asked to rate (on a 5-point Likert scale, with 1 = not at all, 5 = very much) whether they considered the presence of the mannequin “not pleasant” or “pleasant”, “annoying” or “not annoying.” All participants rated the presence of the mannequin pleasant (M = 4.42, SD = 0.67), and not annoying (M = 4.10, SD = 0.68). 

Finally, the whole experimental session lasted about 30–35 min.

## 3. Results

### 3.1. Data Analysis

Preliminary descriptive analyses were performed to assess missing values and variable distributions. Univariate distributions of observed variables were examined for normality and outliers [60]. Data with SD ± 2.5 (corresponding to 0.4% of the data) were excluded from the analysis. The Skewness and kurtosis values of the data indicated that the distribution was normal: Reaching distance, average skewness = 0.508, range: 0.130 to 0.805; average kurtosis = 0.429, range: −0.429 to 1.776; Comfort distance, average skewness = 0.198, range: −0.759 to 0.668; average kurtosis = −0.205, range: −1.318 to 0.165; Differential distance, average skewness = 0.125, range: −0.523 to 0.596; average kurtosis = −0.126, range: −0.881 to 0.586 [61]. 

We carried out a sensitivity analysis [62] to determine the minimum effect size that we could reliably detect with our test. The results showed that with a power = 0.80, α = 0.05, and 62 participants, we could detect a Cohen’s f of at least 0.20, corresponding to a partial eta squared (η^²^_p_) of 0.08.

To test the effect of the tool training on the comfort distance and reaching distance in each condition, the distance at which participants stopped the confederates was recorded. In each distance task block, (Pre-Post Reaching and Pre-Post Comfort), the mean participant-confederate distance (cm) was computed. Data were analysed through two separate 3 × 2 Mixed ANOVAs on the mean reaching distance and the mean comfort distance with Group (3 levels: OnlyT; TM; TObj) as between factor and Session (2 levels: Pre-tool-use and Post-tool-use) as within factor.

Moreover, to further check the robustness of the results, the mean participant–confederate distance obtained (in each block) in the Pre-tool-use session was subtracted from the mean participant–confederate distance obtained, in the same condition, in the Post-tool-use session. The rationale was as follows: positive values signal that an elongation of the two spaces occurred whereas negative values signal that a contraction of the two spaces occurred. These data were analysed through a 3 × 2 Mixed ANOVA with Group (3 levels: OnlyT; TM; TObj) as between factor and Task (2 levels: Reaching and Comfort) as within factor.

Finally, we also tested for sex differences by analysing the data through two different 2 × 2 × 2 × 3 ANOVAs on reaching- and comfort-distance tasks, respectively, with Session (2 levels: Pre-tool-use; Post-tool-use) and Confederate’s sex (2 levels: Male; Female) as within factors and Participant’s sex (2 levels: Male; Female), and Group (3 levels: OnlyT; TM; TObj) as between factors.

Data with SD ± 2.5 (1.6%) were excluded from the analysis. The Bonferroni post-hoc test was used. The magnitude of significant effects was expressed by partial eta-squared (η^²^_p_).

### 3.2. Reaching Task

**Figure 2:**
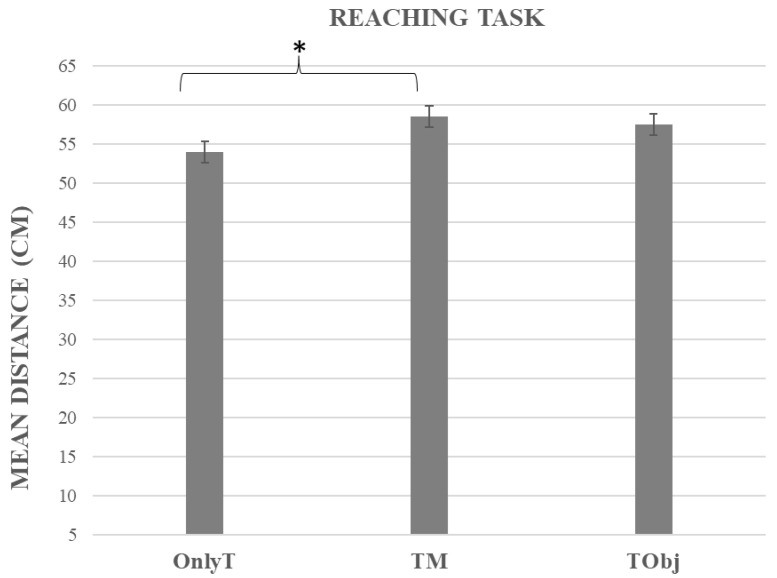
The graph shows the mean reaching distance (in cm) as a function of the three groups (OnlyT: Only Tool; TM: Tool plus Mannequin; TObj: Tool plus Object). The parenthesis and asterisk indicate experimental conditions that differ in approaching significance from each other. Error bars represent the standard error.

The ANOVA showed a significant main effect of the Group (F(2,59) = 3.30, *p* = 0.04, η^2^_p_ = 0.10). The related means were as follows: TM = 58.58 cm, SD = 8.86; TObj = 57.53 cm, SD = 7.85; OnlyT = 54.03 cm, SD = 8.34. The post-hoc analysis showed that the reaching distance was larger in the TM group than in the OnlyT group (approaching significance, *p* = 0.051) but not in the TObj group (*p* = 1) (see Figure 2). No difference emerged between the OnlyT and TObj groups (*p* = 0.20). 

Moreover, a significant main effect of the Session was observed (F(1,59) = 9.16, *p* < 0.01, η^²^_p_ = 0.13). The reaching distance in the Post-tool-use session was larger than in the Pre-tool-use one (Pre = 54.59 cm; SD = 6.80; Post = 58.81 cm; SD = 9.56). See Table 1 for descriptive statistics. 

No significant interaction emerged between Session and Group (F(2,59) = 1.37, *p* = 0.26).

### 3.3. Comfort Task

The ANOVA showed a significant main effect of the Group (F(2,59) = 3.33, *p* = 0.042, η^²^_p_ = 0.10). The related means were as follows: TM = 50.21 cm, SD = 15.33; TObj = 44.30 cm, SD = 15.33; OnlyT = 40.33 cm, SD = 15.06. The post-hoc test showed that the comfort distance in the TM group was larger than the distance in the OnlyT group (*p* = 0.038). No other significant comparison emerged.

**Figure 3:**
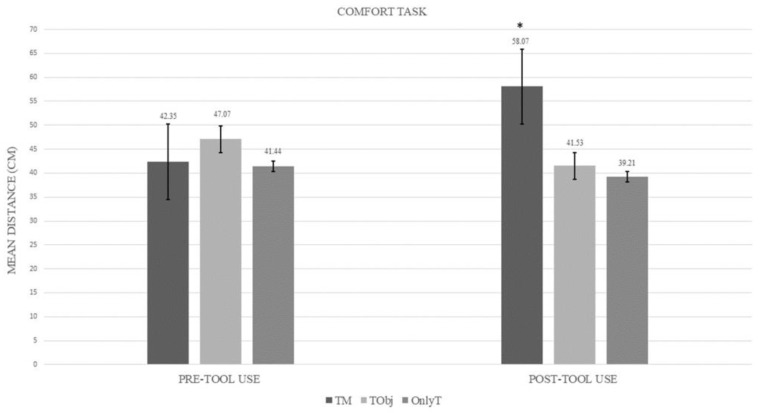
The graph shows the mean comfort distance (in cm) as a function of the three groups (OnlyT: Only Tool; TM: Tool plus Mannequin; TObj: Tool plus Object) and the Pre-tool-use and the Post-tool-use conditions. The asterisk indicates the experimental condition that differs significantly from the others. More specifically, the Post-tool-use distance in the TM group was significantly different from the Post-tool-use distance in the other two groups and from the Pre-tool-use distance in the OnlyT and TM groups. Error bars represent the standard error.

In contrast with the reaching distance task, no main effect of the Session emerged (F(1,59) = 1.90, *p* = 0.17). The related means were as follows: Pre-tool-use = 43.57 cm; Post-tool-use = 46.35 cm. Notably, a significant interaction between Group and Session emerged (F(2,59) = 11.82, *p* = 0.00005, η^²^_p_ = 0.29). The Bonferroni post-hoc test clearly showed that the comfort distance was larger in the Post-tool-use than in the Pre-tool-use (*p* < 0.001) only in the TM group, as shown in Figure 3. Furthermore, in the TM group, the Post-tool-use distance was larger than the Post-tool-use distances in the other two groups (at least, *p* < 0.01), and the Pre-tool-use distance in OnlyT group (*p* = 0.005). Instead, no significant difference emerged between the Pre- and Post-tool-use in the OnlyT group (*p* = 1) as well as in the TObj group (*p* = 1).

### 3.4. Analyses on Differential Distance

**Figure 4:**
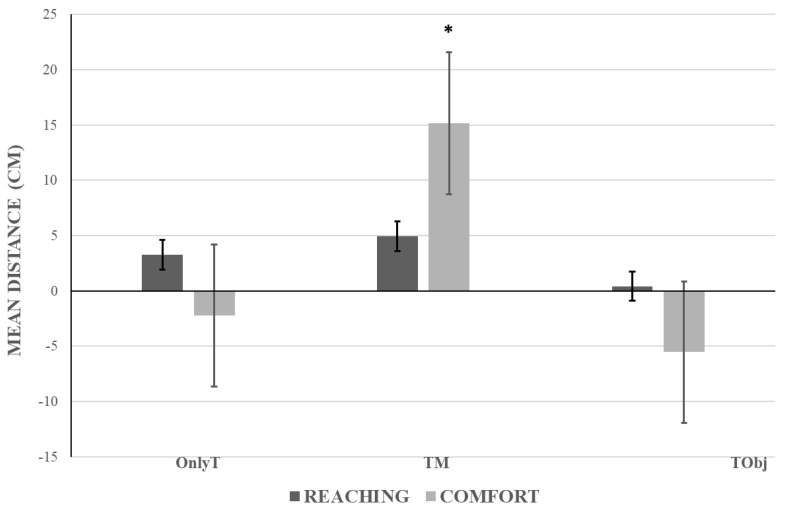
Interaction Group × Task. The graph shows the mean distance (in cm) as a function of the three groups (OnlyT: Only Tool; TM: Tool plus Mannequin; TObj: Tool plus Object) and the reaching- and comfort-distance tasks. The asterisk indicates the experimental condition that differ significantly from all others. More specifically, the comfort distance in the TM group was significantly different from the comfort distance in the OnlyT and TObj groups and from the reaching distance in all three groups. Error bars represent the standard error.

The ANOVA showed a significant main effect of the Group (F(2,59) = 8.73, *p* = 0.0005, η^²^_p_ = 0.23). The related means were as follows: OnlyT = 0.52 cm, SD = 9.76; TM = 10.33 cm, SD = 15.18; TObj. = −2.56 cm, SD = 15.54. In the social context, the presence of the mannequin led to an increase in distance in the TM group compared to the other two groups (at least, *p* < 0.01). There was no significant main effect of Task (F < 1). Furthermore, Group and Task significantly interacted (F(2,59) = 6.79, *p* = 0.002, η^²^_p_ = 0.19) (Figure 4). The post-hoc comparisons showed that the three groups did not differ in terms of reaching distance (*p* = 1). The related means were OnlyT = 3.27 cm, SD = 9.75; TM = 4.95 cm, SD = 13.45; TObj = 0.42 cm, SD = 9.8. The positive values indicate that the elongation of reaching distance occurred in all groups following the tool-use training. The post-hoc analysis showed that the comfort distance was larger in the TM group than the OnlyT (*p* = 0.009) and TObj (*p* = 0.00001) groups. More specifically, while the mean comfort distance in the TM condition increased after the training with the tool (TM = 15.72 cm, SD = 15.19), the mean comfort distance in the OnlyT and TObj conditions decreased (OnlyT = −2.23 cm, SD = 9.18; TObj = −5.54 cm, SD = 19.52).

### 3.5. Sex Differences 

#### 3.5.1. Reaching Task

Here we reported only the statistically significant effects. A main effect of the Group (F(2,56) = 4.06, *p* = 0.02, η²_p_ = 0.13) emerged with the distance in the TObj group being larger than the distance in the OnlyT group (*p* = 0.03). The related means were: TObj = 59.90 cm, SD = 11.65; OnlyT = 53.67 cm, SD = 10.90. A significant interaction between the Confederate’s sex and Group (F(2,56) = 11.75, *p* = 0.00005, η^²^_p_ = 0.29) was found. In the TObj group, the distance with male confederates was larger than with male and female confederates of the OnlyT group (at least, *p* = 0.005). Within the TObj group, the distance with male confederates was larger than with female ones (*p* = 0.0003).

#### 3.5.2. Comfort Task 

Here, we reported only the statistically significant effects. The analysis showed a main effect of the Participant’s sex (F(1,56) = 5.83, *p* = 0.02, η^²^_p_ = 0.094), such that female participants preferred a larger distance than male ones (*p* = 0.008). The related means were as follows: Male participants = 43.34 cm, SD = 19.12; Female participants = 54.66 cm, SD = 23.37.

A main effect of the Group emerged (F(2,56) = 7.93, *p* = 0.001, η^²^_p_ = 0.22). The distance was larger in the TObj group than in the OnlyT (*p* = 0.0005) and TM (*p* = 0.01) ones. The related means were as follows: TObj = 61.22 cm, SD = 25.82; OnlyT = 40.76 cm, SD = 18.27; TM = 45.05 cm, SD = 15.54. 

A main effect of Confederate’s sex emerged (F(1,56) = 12.94, *p* = 0.001, η^²^_p_ = 0.19) with a larger distance with male than female confederates. The related means were as follows: Male = 51.34 cm, SD = 23.26; Female = 46.27 cm, SD = 20.41.

The Confederate’s sex and Group significantly interacted (F(2,56) = 3.98, *p* = 0.02, η^²^_p_ = 0.12). The post-hoc test showed that the distance with male confederates in the TObj group was larger than all other conditions (at least *p* = 0.001).

Participant’s sex, Confederate’s sex, and Group significantly interacted (F(2,56) = 3.61, *p* = 0.03, η^²^_p_ = 0.11). The effect was due to female participants in the TObj group preferring a larger distance from male confederates with respect to all other conditions in the other two groups (at least, *p* = 0.05). Within the TObj group, the female participants preferred a larger distance when interacting with male than female confederates (*p* = 0.003).

## 4. Discussion

In this study, we aimed at better understanding whether the processing of information from the social context combined with motor plasticity induced by tool use may or may not reflect a similar modulation in both reaching and comfort spaces. To this end, participants performed tool-use training sessions under the following conditions: in the presence of a mannequin (determining the social context) (TM), in the absence of any kind of stimulus (OnlyT), and in the presence of a box (TObj, as a control).

Overall, the results showed that the distance in the social condition was quite large compared to the conventional only tool-use training. However, the most important result concerns the different effect of the tool-use training in the two reaching and comfort spaces. Regarding the reaching distance, we hypothesized a larger post-tool-use distance in all training conditions. Confirming our hypothesis, data showed that the reaching distance always increased after using the tool, regardless of whether or not a stimulus was present. Regarding the comfort distance, we hypothesized a larger post-tool-use distance only in the social context condition. In line with the assumption, results confirmed that the comfort distance only increased after using the tool in a social context, that is, in the presence of the mannequin.

As for sex differences, we found that the distance was larger with male confederates, especially for female participants in the TObj group. This is in line with previous data showing that individual spatial behaviour is affected by the valence of stimuli (social vs non-social) and sex, with women increasing their body distance when dealing with an object with no social valence (e.g., [5]). This might be due to their sensitivity for the possibility of communicating and the social meaning of a situation (e.g., [1,6]). 

Two points of our results are worth pointing out. First, the general increase in the distance due to simple tool use gives further support to the motor plasticity attributed to the representation of the space around the body conceived as reaching space (e.g., [63,64,65]).The active use of a long tool extends the representation of the reachable space by ideally expanding the point at which one might act and by confirming a close relationship between the arm action capabilities and reaching space (e.g., [11,18,42,44,45,46,47,63,64,65,66]). Moreover, in line with the existent data, tool use modifies the reaching distance towards a potentially relevant social stimulus (e.g., [39,40]).

Secondly, the clear plastic effect of just using a tool on the space of reaching but not comfort should be consistent with the evidence that these spaces can be dissociable [39,40]. However, here we provide a plausible demonstration that reaching and social spaces could share, at some point, sensorimotor mechanisms, as our results showed that tool-use-dependent plasticity may be elicited, even in a social context. In fact, within the ‘social context’ (i.e., TM group), the comfort distance was larger after tool use than in the Pre-tool-use session, whereas no difference in comfort distance after tool use emerged in the conditions without any stimulus (OnlyT) and in presence of an object (TObj). This means that it is not sufficient to use a long tool to provoke a lengthening of the comfort distance, but that in addition to active training it is necessary to involve a socially connoted context such as a mannequin [4,7,19,30,33,56,58]. However, the lack of difference between TM and OnlyT (although approaching significance, *p* = 0.051) may be strengthened by the accentuation of anthropomorphic features of the social stimulus. Likewise, the findings seem to suggest that, to trigger a tool-use-dependent plasticity of the social space, the specifics of the social context should also be considered. For example, a cooperative tool-use training or a short-tool-use training with a confederate led to a contraction of the social space boundaries (see [39,40]).

Therefore, how can we explain the divergence on motor plasticity of comfort distance from previous studies [39,40]? We remind that what happens during the use of the tool seems to influence the subsequent determining of the reaching and comfort distance with respect to real confederates. Therefore, when interpreting the results, we must take into account two aspects: which variables can influence the determination of reaching/comfort distances and how the tool use takes place. Regarding the former, we kept under control the tendency to reduce comfort distance induced by the increased sense of familiarity by using unfamiliar confederates in Pre- and Post-tool-use sessions [48,49,50,51]. As regards to tool-use, it is important the social value elicited by the stimulus. However, we did not include a cooperative condition with a real person that could have pushed people to reduce comfort distance in order to do not invade the other’s portion of space (e.g., [54,55]). The nature of interpersonal comfort-distance is social by definition, in the sense that it expresses the socio-emotional value of interactions between people through dynamic changes in distance [1,2,31,32]. It is possible that training with the tool in a social context (the presence of a social stimulus) together with the maintenance of a constant comfort level thanks to unfamiliar confederates made it possible to reveal the dynamic aspect of the social space.

## 5. Conclusions

In conclusion, the combination of motor plasticity and social context information processing can modulate the boundaries of the comfort space in a similar way to the reaching space, thus revealing a behavioural similarity rooted in action with conspecifics. However, motor plasticity impacts reaching and comfort spaces to different degrees: while reaching space is markedly sensitive to motor plasticity, comfort space needs social information qualification. However, to what extent reaching and comfort spaces may represent specialized functions and what is the role of social-emotional context processing still deserve further investigation. Moreover, our findings could also reflect the relevance that a social stimulus takes on when it is embedded in one’s social space during the sensorimotor re-encoding of the proximal space due to the active use of a long tool. This would converge with the data that emerged from the research on joint actions and shared spaces [54,55,67,68].

Further, although the results indicate that social space also underlies sensorimotor mechanisms, this study has some limitations that could be addressed by future research. For example, the between-subjects experimental design may not have mitigated potential effects due to individual differences. In fact, as shown by the analyses on participants’ sex differences, it is quite evident that further aspects, as well as aspects of personality, need to be taken into account. In addition, in line with previous studies, an experimental condition in which participants use a short tool (along with a long one) could be added. This additional control condition would reinforce the idea that the plasticity of social space is indeed due to the combination of tool use and information processing of the social context.

From a theoretical point of view, this study may contribute to the debate on the nature of action space and social space [69] by highlighting how the regulation of reaching space in the social context has points of contact with the modulation of interpersonal comfort space. However, further studies are needed to understand to what extent and under what conditions these two spaces may reveal similar behavioural displays. Furthermore, many studies have identified alterations of the representation of the space around the body in neurological [70], psychopathological [64,71,72] or autistic [19] patients. Therefore, in-depth knowledge of the mechanisms of sharing or segregation between action space and interpersonal social space may potentially suggest new ways to improve or rehabilitate the functions of representing the space around our bodies.

## Figures and Tables

**Table 1:** Mean and SD Reaching Distances (cm) of the Pre-tool-use and Post-tool-use conditions for each group.

Groups	Pre-Tool-Use (cm) M (Sd)	Post-Tool-Use (cm) M (Sd)
OnlyT	52.36 (7.2)	55.70 (9.22)
TM	54.92 (6.78)	62.24 (9.31)
TObj	56.58 (5.98)	58.48 (9.42)

## Data Availability

The data presented in this study are available on request from the corresponding author.
